# Targeted therapies in non-small cell lung cancer: present and future

**DOI:** 10.12703/r/12-22

**Published:** 2023-09-04

**Authors:** Jessica McLaughlin, Jonathan Berkman, Patrick Nana-Sinkam

**Affiliations:** 1 Department of Internal Medicine, Division of Pulmonary Disease and Critical Care Medicine, Virginia Commonwealth University Health System, Richmond, VA 23298; 2 Department of Internal Medicine, Division of Hematology, Oncology and Palliative Care, Virginia Commonwealth University Health System, Richmond, VA 23298

**Keywords:** Non-small cell lung cancer, molecular biomarker, targeted therapy, oncogene, driver mutation, precision oncology

## Abstract

Lung cancer is the leading cause of malignancy-related death in the United States and the second most common cancer diagnosis worldwide. In the last two decades, lung cancer treatment has evolved to include advances in the development of mutation-based targeting, immunotherapy, radiation therapy, and minimally invasive surgical techniques. The discovery of lung cancer as a molecularly heterogeneous disease has driven investigation into the development of targeted therapies resulting in improved patient outcomes. Despite these advances, there remain opportunities, through further investigation of mechanisms of resistance, to develop novel therapeutics that better direct the personalization of lung cancer therapy. In this review, we highlight developments in the evolution of targeted therapies in non-small cell lung cancer, as well as future directions shaped by emerging patterns of resistance.

## Introduction

Lung cancer remains the second-most common cancer diagnosis worldwide, afflicting 2.2 million people and accounting for 18% of cancer deaths^1^. In the United States (U.S.), lung cancer continues to be the leading cause of malignancy-associated death at approximately 22%^2^. In 2023, epidemiologists estimate that there will be 238,340 new cases of lung cancer and 127,070 deaths attributed to lung cancer in the U.S. alone^3^. The histopathologic subtypes of lung cancer follow the 2021 WHO classification schema and are largely based on morphology and immunohistochemical (IHC) staining patterns, which impact treatment decision-making and prognosis^4^. The two largest categories of lung cancer are small cell lung cancer (SCLC) and non-small cell lung cancer (NSCLC), which account for 14% and 85% of cases, respectively^5^. Current survival estimates of lung cancer are based on the stage at diagnosis. NSCLC has a 5-year survival rate of 65% in localized disease and 9% in metastatic disease. SCLC has even more dismal odds, with a 5-year survival rate of 30% in localized disease and 3% in metastatic disease^6^. In the last two decades, significant gains have been made in understanding the underlying biomolecular mechanisms that drive cancer initiation and progression, which have led to the discovery of somatic “driver mutations” (oncogenes) which, when present, block the normal regulatory cellular feedback processes that appropriately maintain cell growth and survival. Such driver mutations can transform normal cells into malignant cells characterized by unchecked growth and proliferation. Targeted therapies interrupt these aberrant pathways and have revolutionized the approach to treatment for affected patients with increased advantages of tolerability and effectiveness in advanced disease. In this review, we explore the current standard of care, as well as emerging targeted therapies in clinical trials for those with NSCLC with driver mutations; we do not address advances in chemotherapeutics or immune checkpoint inhibitors (ICIs). Within each section, we provide up-to-date epidemiological data, pathogenic mechanisms, an overview of therapeutic agents, and currently available clinical trials.

## *EGFR* mutations

One of the most well-understood driver oncogenes in NSCLC is the *EGFR* (epidermal growth factor receptor) gene on chromosome 7, which encodes one of the erbB family of cell surface transmembrane receptor tyrosine kinases known as erbB-1 or HER1^7^. Once the tyrosine kinase becomes activated, multiple downstream signaling pathways are triggered, such as the Ras-Raf-MEK-ERK, and PI3K-AKT-mTOR, promoting DNA synthesis and cell proliferation^8^. Dysregulation in these pathways drives tumor proliferation, differentiation, and migration. The frequency of activating mutations in *EGFR* in NSCLC (10–15% prevalence in the US) differs based on histology, smoking status, ethnicity, and sex. These mutations are largely observed in adenocarcinomas, those of Asian descent (22–62%), in non-smokers, and females. Multiple generations of oral *EGFR* tyrosine kinase inhibitors (TKIs) have been approved by the US Food and Drug Administration (FDA) for first-line therapy in those with *EGFR-activating* mutations in advanced NSCLC. This is a result of improved outcomes when compared to standard-of-care platinum doublet-based chemotherapy. It should be noted; however, that *EGFR* amplification or overexpression does not have the same predicted responsiveness to targeted therapies. More recently, testing for *EGFR* mutations is also recommended for those patients with resectable stage IB to IIIA NSCLC to guide consideration of adjuvant or neoadjuvant targeted therapy^9^.

Among those patients with *EGFR* driver mutations, the majority have either an exon 19 deletion (45%) or the exon 21-point mutation L858R (40%). These mutations are the most well-studied in NSCLC, with several FDA-approved therapies available impacting patient survival. The early generation TKIs (gefitinib, erlotinib, and afatinib) delivered excellent success rates in improving median progression-free survival (mPFS) when compared to standard chemotherapy in patients with *EGFR* mutations; however, patients develop disease progression while on therapy^10–18^. Osimertinib was approved by the FDA in 2018 to combat the most common resistance mechanisms, T790M mutation on exon 20 of *EGFR* and exon 21 L858R mutations^19–22^. Validation studies demonstrated superior overall survival rates for patients treated with osimertinib when compared to other *EGFR*-TKI therapies, particularly in those with brain metastases^23,24^. Unfortunately, in clinical use, we have learned that many patients develop resistance to osimertinib, which limits further definitive treatment options. Based on the current understanding of tumor heterogeneity and complex cellular signaling within the tumor and microenvironment and advances in molecular sequencing, we have identified both *EGFR*-dependent activating mutations and *EGFR*-independent mechanisms of resistance^25–27^.

### *EGFR*-dependent activating mutations

A tertiary *EGFR* mutation, C797S, has been identified in the presence or absence of the secondary T790M mutation as playing a role in potential therapeutic options, depending on molecular positioning and availability of the binding subunits^26,28–31^. In vivo studies are investigating the use of brigatinib, which is a next-generation *ALK*-TKI, to target triple mutation (exon 19 deletion, T790M mutation, and C979S activating mutation) *EGFR* NSCLC^32^. Uncommon *EGFR* tertiary activating mutations on exons 18-25 include L718/G719, G796, and L792, especially when the T790M mutation is retained^33,34^, and S768I, S720F, E709K, and L861Q, which are typically found in conjunction with more common EGFR mutations^35^. Erlotinib has shown disease stability, albeit short-lived, in a subset of these patients with S786I mutations^36^. Afatinib is the only FDA-approved therapy for rare EGFR mutations, including S768I, L861Q, or G719X^37–39^. There are small signals that osimertinib has activity against these uncommon mutations in EGFR-positive NSCLC, even in patients with brain metastases^40,41^.

Exon 20 insertion mutations have also been discovered as potential targets for individualized therapies^42,43^. Due to the location on exon 20, the insertion mutation has similar properties as *ERBB2 (HER2),* although more heterogeneous in nature, making it difficult to systematically identify targetable features. Due to the *ERBB* inhibitory activity, poziotinib has been studied in vitro with good activity against exon 20 insertion mutations^44^. However, in early clinical studies, there are mixed results with concern for significant adverse effects of skin and gastrointestinal toxicities, which have required significant dose reduction^45,46^. In July 2022, Elamin *et al.* published a study of 50 patients with *EGFR* exon 20 point mutation-positive NSCLC who underwent treatment with poziotinib and achieved the primary-end point of an objective response rate (ORR) of 32% and mPFS of 5.5 months; however, clinical activity varied depending on near or far loop insertion of the point mutation^47^. Mobocertinib received FDA approval for subsequent breakthrough therapy in 2021 for patients with exon 20 insertion mutations after Riely *et al.* showed a confirmed response rate of 43%, median duration of response (mDOR) of 14 months, and mPFS of 7.3 months in 28 patients with exon 20 insertion mutations^48,49^. Unfortunately, the data was most promising in those without brain metastases. Significant side effects of mobocertinib included gastrointestinal side effects in up to 82% of patients with exon 20 insertion mutations. In December of 2021, in an open-label phase I/II study, Zhou *et al.* confirmed a durable clinical benefit for patients with previous platinum-based therapy who were treated with mobocertinib achieving an ORR of 28%, durable clinical response (DCR) of 78% and duration of response (DoR) of 17.5 months with mPFS of 7.3 months^50^. Amivantamab (JNJ-6118372) is a new fully human *EGFR*-*MET* antibody with an immune cell-directing activity that targets *EGFR* mutations, as well as *MET* mutations and amplifications. It inhibits ligand binding and promotes a receptor-antibody complex resulting in antibody-dependent cellular toxicity and death^51^. It received FDA approval for breakthrough therapy in patients with NSCLC and exon 20 insertion mutations who have already received platinum-based chemotherapy based on the CHRYSALIS study after achieving an ORR of 40%, including a complete response in 3 patients, a mDoR of 11.1 months, and mPFS of 8.3 months^52^. Side effects of amivantamab are usually less severe, citing rash as the most common side effect (86%), but only 4% of patients experienced severe effects. There are multiple ongoing studies testing these novel antibody mechanisms for new therapies, including the comparison of targeted therapies to the current standard of care in this rare population with limited available treatment options (NCT04129502, NCT04209465, NCT04538664)^53–55^. Due to significant clinical effect in other subpopulations, including those with brain metastases, osimertinib is also under investigation for patients with exon 20 insertion mutated EGFR positive NSCLC.

### EGFR-independent acquired resistance mechanisms

Loss of the secondary T790M mutation usually results in *EGFR-independent* resistance mechanisms, which can occur via multiple pathways. Some of the most recognized to date include *MET* amplification, *KRAS* mutations, and *BRAF* mutations, among others^33,34,56,57^. *MET* amplification has been identified as the second most common mutation for resistance to the current *EGFR*-TKI therapies^25,26,33,34^. There are preclinical studies evaluating the use of combination *EGFR/MET* inhibitors in addition to third-generation *EGFR*-TKIs to overcome resistance with encouraging results in clinical trials in favor of combination therapy over chemotherapy^58–60^. Although *BRAF* is known as a primary oncogenic driver in NSCLC, it has also been implicated as a mechanism of resistance to *EGFR*-TKI therapy^61–63^. In preclinical studies, Jeong *et al.* discovered *EGFR* and *BRAF* fusion as a mechanism of resistance to the third-generation *EGFR*-TKI, lazertinib^27^. Combination therapy with lazertinib and a *MEK* inhibitor showed strong anti-tumor activity, suggesting a promising therapeutic option^27^. Some less common but still identifiable resistance mechanisms include *HER2* amplification, *TP53* mutation, *KRAS* mutation, induced epithelial-mesenchymal transformation (EMT), *ALK* resistance, and histologic transformation, including conversion to small cell carcinoma^26,47,64–67^.

### Additional ERBB family mutations (*HER2* mutations)

While *HER2* mutations are not the next most common primary mutations in NSCLC, they are part of the *ERBB* receptor tyrosine kinase family and are studied in EGFR mutant NSCLC. *HER2* mutations are present in 2–4% of NSCLC and typically expressed in adenocarcinoma among never smokers and women with a reported incidence of 20.4%^68–70^. *HER2-positive* status in NSCLC appears to confer resistance to a standard regimen of platinum-based chemotherapy^70,71^. There are three categories of inhibitors, including pan-*HER* TKIs, anti-*HER2* monoclonal antibodies, and anti-*HER2*/*HER3* antibody-drug conjugates, that have been used to investigate potential targeted treatment options for those with NSCLC^72,73^. Patritumab (*HER3*-DXd) is an antibody-drug conjugate consisting of a *HER3* antibody attached to a topoisomerase I inhibitor. This molecule has been studied in Phase I trials in patients with *EGFR*-mutated NSCLC who developed resistance to *EGFR*-TKI therapy, with an ORR of 39% and mPFS of 8.2 months^74^. Poziotinib and afatinib showed mixed results in a study of 7 patients, mostly consisting of women non-smokers with stage IV adenocarcinoma and no coexisting *EGFR* or *ALK* mutations^68^. The NICHE study reported mixed results in which afatinib achieved disease control in less than 50% of the study participants at 12 weeks^75^. In 2021, Li *et al.* reported that trastuzumab deruxtecan achieved ORR of 55%, mDoR of 9.3 months, mPFS of 8.2 months, and median overall survival (mOS) of 17.8 months in patients with *HER2*-mutated NSCLC (DESTINY-Lung01)^76^. The authors of the DESTINY-Lung 02 trial reported a confirmed ORR of 56% and mDOR of 8.7 months, which gained trastuzumab deruxtecan accelerated approval by the FDA in August 2022 for patients with *HER2*-mutated NSCLC^77^. While there was an adequate safety profile, a select number of patients developed interstitial lung disease, resulting in 2 deaths^76^. More investigation is required to determine additional therapy options.

Studies are ongoing to investigate the emerging resistance patterns of *EGFR*-mutant NSCLC and new potential therapies^78–81^. Although many molecular mechanisms of resistance to *EGFR*-TKI therapy have been identified, our understanding of how these mechanisms impact therapeutic effects is incomplete.

## *RAS* mutations

The *KRAS* proto-oncogene drives several hallmarks of cancer, including cellular proliferation, differentiation, and survival. *KRAS* mutations are commonly found in about 30% of patients with adenocarcinoma^82–84^. However, selective targeting of this mutation remains a significant challenge due to multiple downstream signaling pathways. The most common *KRAS* mutation is *KRAS*-G12C which is present in about 13% of lung adenocarcinomas. In August of 2020, Jänne *et al.* reported the success of adagrasib in the KRYSTAL-1 study, where 45% of 51 patients with NSCLC had an objective response and DCR of 96% with mDoR of 8.5 months, which ultimately led to FDA approval of adagrasib in December 2022^85^. Preclinical animal model data supports the need for ongoing studies for the use of adagrasib in brain metastases^86^. In May of 2021, the FDA-approved sotorasib, a *RAS* GTPase inhibitor, for treatment in those with *KRAS* G12C mutated locally advanced or metastatic NSCLC who have received at least one prior systemic therapy^87^. Approval was based on the results of the Codebreak 100 trial, which showed significant responses (37% ORR and 3.2% of patients achieving complete response) with a favorable safety profile and mPFS of 6–8 months^87–89^. As with other kinase inhibitors, some patients are now developing resistance with evidence of disease progression while on therapy. The mechanisms of resistance remain incompletely understood. Awad *et al.* described 38 patients with *KRAS* G12C mutations, 10 of whom had colorectal cancer, 1 with appendiceal cancer, and the remaining 27 with NSCLC, who all progressed on adagrasib after 12 weeks^90^. 84% of the patients maintained the original *KRAS* G12C mutation while on therapy. The remaining 16% had no detectable *KRAS* G12C mutation in plasma after therapy. Mechanisms of resistance included novel secondary *KRAS* mutations (Y96C, H95Q, H95R, R68S, H95D) in four patients, in addition to *KRAS*-activating mutations (G12D, G12V, G13D, G12W)^90^. Pathogenic mutations in other receptor tyrosine kinase (RTK)-RAS MAPK pathways were also detected, including NRAS, *BRAF*-V600E, MAP2K1/*MEK*1, *EGFR*, histological transformation, and high-level focal amplification in the *KRAS* G12C allele, in addition to acquired *MET* amplification^90,91^. PI3K-AKT has also been implicated as a likely resistance pattern for *KRAS*-targeted therapies^92^. Deep mutational scanning was used to identify all possible alleles with a single amino acid substitution within *KRAS* G12C. Novel drug resistance mutations were identified at codons 8, 9, 12, 64, 68, 95, 96, 99, and 117 with strong resistance to *KRAS* G12C inhibitors in addition to the known adagrasib-resistant *KRAS* mutations, which were also resistant to MRTX1257 (sotorasib analog)^90^. There were also novel strong resistance mutations to sotorasib at codons 8, 9, 12, 96, and 117^90^. Most were commonly found to occur at the drug-binding site; however, there were amino acids located outside of the drug-binding pocket that impeded GTP hydrolysis (G13D and Q16R) or facilitated GDP to GTP nucleotide exchange (G13D, A59S, and A146P)^90^. Several highly selective *KRAS* G12C inhibitors are in development, including those that bind to the active GTP bound conformation, which in theory, increases the inhibitory activity and promotes control (RMC-6261)^91^. Epithelial mesenchymal transition (EMT) has been shown to lead to both acquired and intrinsic resistance to *KRAS* G12C inhibition with multi-targeted therapy showing tumor regression in mice models^93^. There are studies underway to identify additional targets in these pathways^94,95^. In addition to the approval of sotorasib and adagrasib, there are many more *KRAS* G12C pathway inhibitors under investigation^83,96–101^. There are also active studies of the precursor signals that activate *KRAS* and contribute to cell survival and proliferation, like Son of Sevenless (SOS1) and SHP2, which have the potential to overcome acquired resistance to *KRAS*-targeted therapies^102–108^.

## *MET* mutations or amplification

*MET* alterations, most commonly skip mutations, occur in 3–4% of NSCLC, and drive tumor proliferation, invasion, and metastasis^10^. A nonselective *MET* inhibitor, crizotinib achieved an ORR of 32% and mDoR of 9.1 months with PFS of 7.3 months and has been validated in follow-up phase II studies in patients with *MET* exon 14 mutations^109,110^. Crizotinib was approved for breakthrough therapy in this subclass of patients by the FDA in 2018. In a phase II study, Wolf *et al*. proved that capmatinib exhibited an ORR of 41% in 69 patients who had previously received therapy and an ORR of 68% in treatment naïve patients^111^. The mDoR was 9.7 months and 12.6 months, respectively. There was less of a response in patients with *MET* amplification mutations, with an ORR of 29% and 40% for those who were previously treated and treatment naïve, respectively^111^. There are phase II studies reporting activity and response with tepotinib, savolitinib, and capmatinib in NSCLC patients harboring *MET* exon 14 skip mutations^111–113^. The only FDA-approved *MET*-targeted therapies include capmatinib, which was approved in May 2020, and tepotinib, approved in February 2021^114,115^. Phase III trials are ongoing comparing capmatinib to chemotherapy in those with *MET* exon 14 skip mutations in NSCLC^116^. The mechanisms of resistance to early-generation *MET*-TKIs are not fully understood but found to include many of the same resistance mechanisms described in *EGFR*-positive NSCLC. Recondo *et al.* studied 20 patients and identified genetic alterations or bypass signaling in 15 of those patients, which included single and polyclonal *MET* kinase domain mutations at codons H1094, G1163, L1195, D1228, Y1230, high levels of *MET* amplifications on exon 14, *KRAS* mutations and amplifications, *EGFR*, *HER3*, and *BRAF*^117^. There are small case reports of *BRAF* V600E mutation leading to resistance to crizotinib^118^.

## *BRAF* mutations

BRAF is a *RAS*-activated intracellular serine/tyrosine kinase protein. *BRAF* mutations are identified in about 2–5% of NSCLCs, resulting in persistent downstream signaling of mitogen-activated protein kinase (MAPK), driving tumor growth and proliferation^10^. Activating V600E mutations comprise half of the cases of *BRAF* mutations^119,120^. Resistance to therapy emerges through *MEK* 1 and 2 signaling pathways^121–123^. There are three types of targeting inhibitors under investigation (*BRAF* inhibitors, *MEK 1* and *2* inhibitors, and *ERK* inhibitors). *BRAF* inhibitor therapy alone has little effect as monotherapy^121,124^. However, the *BRAF* inhibitor (dabrafenib) plus *MEK1* and *2* inhibitor (trametinib) demonstrate good activity in phase II clinical trials with an ORR of 63% and mDoR of 9 to 15 months^125–127^. The *BRAF* inhibitor vemurafenib demonstrates activity among those with *BRAF*-V600E mutations exhibiting ORR between 37–44% and mPFS of 6.5 months^128,129^. Currently, the only FDA-approved regimen for patients with *BRAF* V600E mutations in NSCLC is the combination of two agents, trametinib and dabrafenib^130^. Co-occurring mutations most commonly include *TP53* in small sample sizes^131^. Unfortunately, very little is known about the resistance mechanisms to *BRAF* inhibitor therapy^132^. There are multiple drugs under investigation for the treatment of *BRAF*-mutant NSCLC^133–136^.

## *ALK* rearrangements and *ROS1* rearrangements

*ALK* fusion mutations drive tumor cell proliferation and survival and are present in 4–5% of adenocarcinomas^137–139^. *ROS 1* is a kinase receptor with significant structural similarity to *ALK*^10^. Rearrangements in *ROS 1* can develop into fusions of the *ROS* tyrosine kinase domain with other genes which occur in about 1–2% of NSCLC^121^. There are two main inhibitors associated with *ROS*, those that inhibit *ROS1* and *ALK* and those that inhibit *ROS1* and tyrosine receptor kinase (*TRK*). Crizotinib, in addition to *MET* inhibition, also inhibits *ALK*/*ROS1,* which was FDA approved in 2011 after early studies showed promising results for patients with fusion mutations and validated in later phase III studies^140–144^. To date, the *ALK* inhibitors ceritinib, alectinib, and brigatinib show improved ORR and mPFS when compared to either standard chemotherapy or crizotinib alone in treatment naïve patients^145–147^. Lorlatinib is a selective *ALK*/*ROS1* TKI with brain penetration. In 2020, Shaw *et al.* compared lorlatinib to crizotinib and discovered improved overall survival (OS) at 12 months in the lorlatinib group – 78% to 39%, respectively, an ORR of 76% and 58%, respectively, and brain metastasis response rate of 82% in lorlatinib and 23% in crizotinib^144^. Lorlatinib received FDA approval for a breakthrough therapy for a DCR rate of 50% overall, including intracranial response in those with brain metastases. In 2021, Camidge *et al.* reported the benefits of brigatinib as first-line therapy in patients with *ALK* inhibitor naïve -*ALK*-positive NSCLC, with superior 3-year PFS versus crizotinib, and increased survival in those with brain metastases^148^. *TP53* mutations are the most common genomic co-alteration in *ALK*-positive NSCLC, with overall worse prognosis and survival, with preclinical studies testing ixazomib (a proteasome inhibition), which induced apoptosis in previously alectinib resistance cells^149^. Known mechanisms of resistance to *ALK*-TKI therapy include secondary *ALK* mutations, *BRAF*-V600E mutations, and a novel discovery of epithelial-mesenchymal transition (EMT) of newly metastatic cells^150^. Three additional *ROS1*/*TRK* inhibitors have been studied in *ROS1*-rearranged NSCLC. Entrectinib is a highly potent ATP-competitive TKI that penetrates the central nervous system. Multiple studies show an ORR of 67.1%, DoR of 15.7 months, and mPFS of 15.7 months in 161 TKI naïve patients^151^. Repotrectinib and taletrectinib are *ROS1*/*TRK* inhibitors that can penetrate the CNS. The first in-human phase I study of taletrectinib achieved an ORR of 33.3% in those with critzotinib resistance *ROS*1 mutations^152^. Repotrectinib has shown activity against *ROS1*-resistance mutations and there are ongoing studies to confirm anti-tumor activity^153^. Entrectinib is the only *ROS1*/*TRK* inhibitor to be approved by the FDA in 2019^151,154,155^.

## *RET* rearrangements

The *RET* gene encodes a cell surface tyrosine kinase receptor that impacts cell proliferation and differentiation. In 1–2% of those with NSCLC (most often in adenocarcinomas, never-smokers, and younger patients), rearrangements between *RET* and *CCDC6* (coiled-coil domain containing-6), *KIF5B* (kinesin family 5B), *NCOA4* (nuclear receptor coactivator 4), or other domains ultimately results in dysregulated signaling and *RET* overexpression. Testing via next-generation sequencing (NGS) (RNA-based preferred over DNA-based testing) is the preferred method. Break-apart FISH (fluorescence in situ hybridization) probes or RT-PCR (reverse transcription polymerase chain reaction) can be used; however, they may not detect less common fusion products. Regardless of performance status, first-line systemic therapy for those patients with advanced NSCLC (even with brain metastases) and a *RET* fusion is often with either of the oral *RET* TKIs (selpercatinib or pralsetinib), especially since current evidence is limited and suggests mixed efficacy of immune checkpoint inhibitor monotherapy with an ORR of 6%^156–158^. The landmark phase I/II multi-cohort open-label trial that led to FDA approval for selpercatinib was LIBRETTO-00, where patients were either treatment-naïve or previously received platinum-based chemotherapy for their RET fusion NSCLC. In the 69 treatment-naïve patients, an ORR of 84%, mPFS of 22 months, and mDoR of 20 months were reported, whereas the 247 previously treated patients had an ORR of 61%, mPFS of 25 months, and mDoR of 29 months^159,160^. Among those patients with brain metastases, 26 had baseline measurable disease and were without any radiation therapy in the two months preceding trial enrollment, and 22 patients had an intracranial response (ORR of 85%) with mDoR of 9.4 months; this was consistent whether prior local or systemic treatments were received^160,161^. These rearrangements result in ligand-independent signaling, which leads to oncogenic proliferation and has been implicated in the pattern of resistance to osimertinib^121^. Small studies investigating multi-kinase inhibitors like cabozantinib and vandetinib have reported ORR of 28% and 47%, respectively, in NSCLC^162,163^. In 2020, more selective *RET* inhibitors such as pralsetinib demonstrated promising results achieving ORR of 65%, time to response of 1.8 months, and mDoR not being met^164^. Selpercatinib was tested in 105 patients with *RET* fusion-positive NSCLC and achieved an ORR of 64% and DoR of 17.5 months^159^. The results were superior for treatment-naïve patients achieving an ORR of 85%, and those with nervous system symptoms with an intracranial response rate of 91%^159^. There are ongoing studies under investigation for new selective *RET* inhibitors^165–168^.

## *NTRK* fusions

There are several *NTRK* genes currently identified. These genes serve to encode proteins TRKA, TRKB, and TRKC, which facilitate growth factor receptor binding to induce tyrosine kinase activity, potentiating a cellular signaling cascade^168–170^. *NTRK* fusion genes in malignancy were identified back in the 1980s; however, their role in NSCLC specifically is more novel. Current meta-analyses and systematic reviews detail the prevalence to be between 0.10–0.25% in NSCLC patients^171–173^. NGS is the preferred method for identification of these extremely rare fusion genes^174^. The first generation NTRK-TKI, arotrectinib is a highly selective ATP competitive inhibitor of tropomyosin receptor kinases (TRK), received FDA approval in 2018 for adult and pediatric patients with solid tumors and *NTRK* fusion genes without any additional therapy options^175,176^. Its efficacy was further solidified in a phase I dose escalation study of 70 patients, 8 of whom had TRK gene fusions, and the ORR was 100%^177^. With overall response rates of 75% in a study including adults and children with 17 unique TRK fusion-positive tumors^178^, overall adverse events have been reported to be mild. Entrectinib is an oral selective inhibitor of *NTRK* proteins, *ROS 1*, and *ALK* tyrosine kinase, which received FDA approval in 2019 after Marcus *et al.* proved a DoR rate of 57%, including a complete response of 7% amongst 54 patients with 10 different NTRK fusion-positive malignancies^179^. There are multiple phase I and II trials under investigation for entrectinib, which include pediatric and adult patients with solid tumors and NTRK fusion genes. 10 patients with NSCLC were included and achieved an ORR of 70%^180^. To date, there are select ongoing trials comparing targeted therapies for patients with NTRK-positive NSCLC (NCT04302025, NCT04996121, NCT05192642)^181–183^. 

## Targets on the horizon

Given the molecularly complex nature of NSCLC and potential targeted therapies, the field is evolving rapidly. There are new targets being identified and studied continuously. There are numerous ongoing studies to combine known targeted therapies as adjunct therapy to other modalities, including chemotherapeutics, ICIs, radiation therapy, and surgical resection. The details of these studies are beyond the scope of this review. For a more complete list of molecular targeted therapies under investigation at the time of this publication, we have included ongoing clinical trials for reference in Table 1.

**Table 1.** Targetable Mutation therapy, known resistance mechanisms, and ongoing clinical Trials.

Mutation Target	Prevalence of mutation in NSCLC	Approved Therapies (FDA Approval Year)	Objective Response Rate (ORR)	Known Resistance Mechanisms	Ongoing clinical studies*
EGFR	10–15%	Gefitinib (2003) Erlotinib (2004) Afatinib (2013) Osimertinib (2018) Amivantamab (2020) Mobocertinib (2021)	40–80%	Exon 20 T790M insertion; C797S mutation; L718/G719, G796, L792, S768I, S720F, E709K, L861Q activation, Exon 20 insertion mutations, MET amplification, KRAS mutations, BRAF mutations, HER2 amplification, TP53 mutation, EMT, ALK resistance, histologic conversion	NCT03944772; NCT05681780; NCT05435274; NCT04619004; NCT05338970; NCT04285671; NCT04676477; NCT05068024; NCT03260491; NCT05394831; NCT02438722; NCT04993391; NCT04077463; NCT04538664; NCT04487080; NCT04606771; NCT02414139; NCT05374603; NCT05767892; NCT03778229; NCT05546866; NCT03865511; NCT05430386; NCT04129502; NCT05668988; NCT03515837; NCT05801029; NCT05338619; NCT05498389
KRAS	13–30%	Sotorasib (2021) Adagrasib (2022)	37%	Novel KRAS mutations (Y96C, H95Q, H95R, R68S, H95D), Activating mutations (G12D, G12V, G13D, G12W), NRAS, BRAF, MAP2K1/MEK1, EGFR, MET amplification, histological transformation, PI3K-AKT, EMT	NCT04916236; NCT04449874; NCT04699188; NCT04585035; NCT04111458; NCT04625647; NCT04330664; NCT05054725; NCT05254184; NCT05374538; NCT04685135; NCT04735068; NCT05132075; NCT05451056; NCT05504278; NCT04967079; NCT05375084; NCT01933932; NCT05400577; NCT05789082; NCT04613596; NCT05276726; NCT05472623; NCT03095612; NCT03299088; NCT03520842; NCT02642042; NCT05815173; NCT04933695; NCT05074810; NCT05631574; NCT02743923; NCT05180422; NCT05313009; NCT05118854; NCT02152631
MET	3–4%	Crizotinib (2018), Capmatinib (2020), Tepotinib (2021)	32–41%	MET domain mutations, KRAS mutations, EGFR, HER3, BRAF mutations	NCT04427072; NCT05777278; NCT04084717; NCT05782361; NCT04677595; NCT03539536; NCT04258033; NCT05642572; NCT05435846; NCT05513703; NCT04923945; NCT04739358; NCT04052971; NCT02609776; NCT04077099; NCT04928846; NCT04270591; NCT05009836; NCT05015608; NCT04926831; NCT02929290; NCT03175224; NCT05488314; NCT05652868
BRAF	2–5%	Vemurafenib and Dabrafenib (2019)	37–63% depending on the drug	MEK1/2 signaling	NCT05065398; NCT04526782; NCT05195632; NCT04620330; NCT05641493; NCT02974725; NCT04452877; NCT04913285; NCT04439292; NCT05786924; NCT05275374; NCT02407509; NCT03915951; NCT05355701; NCT05501912; NCT04543188; NCT04190628; NCT04892017
ALK/ ROS1	4–5%/1–2%	Crizotinib (2011), Lorlatinib (2018), Entrectinib (2019)	67–76%	Secondary ALK mutations, BRAF mutations, EMT	NCT01639508; NCT03093116; NCT01970865; NCT04322890; NCT05055232; NCT04237805; NCT02927340; NCT05765877; NCT03607188; NCT04042558; NCT05441956; NCT04621188; NCT03439215; NCT04777084; NCT04989322; NCT02321501; NCT03917043; NCT05845671; NCT04362072; NCT05200481
HER2	2–4%overall. More prevalent in young women who are never smokers with adenocarcinoma, 20%	Trastuzumab deruxtecan (2022)	58%	Unknown	NCT05411276; NCT05016544; NCT03974022; NCT05751018; NCT05141786; NCT05650879; NCT05099172; NCT05048797; NCT05364073; NCT04311034; NCT04818333; NCT05532696; NCT05246514; NCT05745740; NCT04886804; NCT04447118; NCT05013554; NCT03318939; NCT04144569; NCT05653752; NCT02675829; NCT05805956; NCT03066206; NCT03740256; NCT03602079; NCT05786716; NCT05057013; NCT04278144; NCT03821233; NCT02892123; NCT05150691; NCT04319757
RET	1–2%	Selpercatinib (2020); Pralsetinib (2020)	61–85%	Unknown	NCT01639508; NCT03157128; NCT04194944; NCT04222972; NCT04268550; NCT03037385; NCT05117658; NCT05675605; NCT03780517; NCT05278364; NCT04161391; NCT05451602; NCT05241834; NCT04280081; NCT04143711
NTRK	<0.5%	Larotrecitinib (2018); Entrectinib (2019)	70–100% (8–17 patients in each study only 2 studies)	Unknown	NCT04302025; NCT01639508; NCT02920996; NCT04996121; NCT02568267; NCT03093116; NCT02576431, NCT02097810

* Updated as of 4/28/2023

## Conclusions

The discovery of genetic drivers in cancer development, growth, and metastases has individualized cancer treatment. The field continues to evolve rapidly, with numerous ongoing studies examining multiple combinations of therapies and mechanisms of resistance, which opens the door to additional targeted therapies. With this rapidly evolving landscape, special attention must be paid to what therapies have molecular benefits and exploration to ensure these benefits translate to true clinical outcomes for patients.
